# Characterising the interspecific variations and convergence of gut microbiota in *Anseriformes* herbivores at wintering areas

**DOI:** 10.1038/srep32655

**Published:** 2016-09-07

**Authors:** Yuzhan Yang, Ye Deng, Lei Cao

**Affiliations:** 1 School of Life Sciences, University of Science and Technology of China, Huangshan Road, Hefei, 230026, China; 2 Research Center of Eco-Environmental Sciences, Chinese Academy of Sciences, Beijing, 100085, China

## Abstract

Microorganisms in vertebrate guts have been recognized as important symbionts influencing host life. However, it remains unclear about the gut microbiota in long-distance migratory *Anseriformes* herbivores, which could be functionally important for these wetland-dependent animals. We collected faeces of the greater white-fronted goose (GWFG), bean goose (BG) and swan goose (SG) from Shengjin Lake (SJL) and Poyang Lake (PYL) in the Yangtze River Floodplain, China. High-throughput sequencing of 16S rRNA V4 region was employed to depict the composition and structure of geese gut microbiota during wintering period. The dominant bacterial phyla across all samples were *Firmicutes, Proteobacteria* and *Actinobacteria*, but significant variations were detected among different goose species and sampling sites, in terms of α diversity, community structures and microbial interactions. We found a significant correlation between diet and the microbial community structure in GWFG-SJL samples. These results demonstrated that host species and diet are potential drivers of goose gut microbiota assemblies. Despite these variations, functions of geese gut microbiota were similar, with great abundances of potential genes involved in nutrient metabolism. This preliminary study would be valuable for future, exhaustive investigations of geese gut microbiota and their interactions with host.

The last decade has witnessed rapid development in the investigation of the community composition and structure of vertebrate gut microbiota and their interactions with host^1^,^2^. These advances are due to the development of new technique, particularly the 16S rRNA high-throughput sequencing. The crucial functions performed by these microbes on behalf of their host also have been revealed, showing that gut microbiota may perform important roles in regulation of metabolism, immune defence, and even normal host development^3^,^4^.

Birds harbour diverse microbes within their guts just like other vertebrates, with the dominant phyla of *Firmicutes, Proteobacteria, Actinobacteria* and *Bacteroidetes*^5^,^6^. However, the gut microbial community structures of birds differ significantly from those of other vertebrates^7^. These differences may reflect the distinctive morphological characteristics and high-energy requirements involved in supporting the powers of flight in birds^5^. For example, avian species are generally characterised by short gastrointestinal tracts, high body temperature and short food retention time^8^. Recent attention mostly focuses on commercially important species (turkeys^9^, chickens^10^) and endemic wild species ((krill-feeding seabirds^11^ and penguins^12^, scavenging vultures^13^, leaf-eating hoatzins^14^, and the critically endangered kakapo^15^).

However, little is known about the gut microbiota of herbivorous geese, even though the herbivorous diet of wild *Anseriformes (Anatidae*) is unique among bird lineages^16^. A previous study of the gut microbiota in the Canada goose *Branta canadensis* conducted by clone library sequencing was probably incomplete due to the small numbers of both samples and sequences^17^. Recently, Wang *et al*. used high-throughput sequencing of 16S rRNA to compare the gastrointestinal microbial communities of the bar-headed goose *Anser indicus* under three distinctive breeding strategies^18^. However, comprehensive study of different goose species is lacking, particularly of the numerous geese during the energetically and nutritionally challenging wintering periods^19^. In order to understand the potential functions of gut microbes in these geese, we undertook a comparative investigation of the patterns of diversity and community composition amongst the gut microbiota in different goose species.

Three long-distance migratory and wetland-dependent *Anseriformes* herbivores were considered in this study, including the greater white-fronted goose *Anser albifrons*, bean goose *Anser fabalis* and swan goose *Anser cygnoides*. In contrast to the increasing trend in South Korea or Japan, the abundance of these geese is shrinking in China, especially the swan goose which has been listed as Vulnerable by IUCN^19^. This situation may relate to diet selection which is considered to be one of the most important factors influencing the abundance and distributions of waterbirds^20^. In South Korea or Japan, wintering geese mostly feed on agricultural land; while in China, their counterparts still inhabit natural lakes and associated wetlands relying on *Carex* meadow and subterranean tubers^21^,^22^,^23^. Changes in geese population dynamics in China may result from deterioration of these natural habitats and reduction of suitable food resources^21^,^22^. However, the detailed mechanism of geese’s diet selection remains unclear^22^. As gut microbiota may co-evolve with diet selection^24^, analysing these microbes may help us understand geese’s preference of natural food in China and provide new perspectives for geese conservation^25^. Nevertheless, little is known about the gut microbiota or their functions in geese. This study may be an early attempt to examine the gut microbiota of wintering, wild geese under natural dietary conditions in the Yangtze River floodplain that provides the basis for future comparative studies with the same species which are confined to agricultural habitats in other parts of the northern hemisphere.

We included all three species from Shengjin Lake and Poyang Lake during the wintering period to document similarities and differences in goose gut microbiota. High-throughput sequencing of 16S rRNA V4 region and a series of statistical analyses were performed to (i) describe microbial community structure and composition; (ii) identify the drivers of gut microbiota assemblies; (iii) elucidate the potential functions of these microbes.

## Results

### Overview of gut microbioal structures of the herbivorous geese

In total, 73 faeces from the three goose species were collected in January 2015. Twenty-two samples were collected from the greater white-fronted geese at Shengjin Lake (GWFG-SJL), as well as 19 from the greater white-fronted geese (GWFG-PYL), 18 from the bean geese (BG-PYL) and 14 from the swan geese (SG-PYL) at Poyang Lake. Sequencing of the 16S rRNA V4 region generated a database of 3,311,834 reads. After basic processes and a series of quality filtering, 2,766,804 high-quality sequences were assigned to 15,511 operational taxonomic units (OTUs) at a level of 97% similarity using the UPARSE pipeline. Sequences classified as *Archaea, Chloroplast*, and *Mitochondria* were excluded from subsequent analysis, retaining 14,499 OTUs. Although not all the OTU-level rarefaction curves reached the saturation level (Fig. S1a–d), the Shannon index rarefaction curves plateaued in all samples (Fig. S1e–h), indicating that most of the microbial diversity in these faecal samples had already been captured at the current sequencing. The other undetected rare species would not affect our conclusions based on the diversity-indexes.

**Figure 1:**
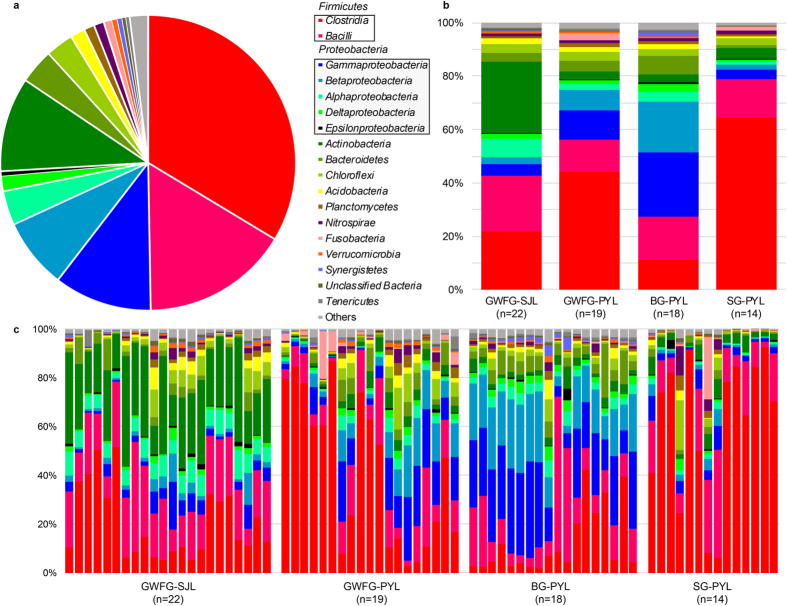
Phylum-level gut microbiota composition of herbivorous geese. (**a**) Relative contribution of the dominant phyla in all samples. (**b**) Relative abundance of these taxa in the four sample groups. Samples are grouped according to sampling location and species. (**c**) Relative abundance of these taxa in each sample. GWFG-SJL refers to 22 samples of greater white-fronted goose at Shengjin Lake. GWFG-PYL refers to 19 samples of greater white-fronted goose at Poyang Lake. BG-PYL refers to 18 samples of bean goose at Poyang Lake. SG-PYL refers to 14 samples of swan goose at Poyang Lake.

Nearly all reads were assignable to 67 phyla, while most were rare. The most dominant phyla were *Firmicutes* (49.70%) and *Proteobacteria* (23.80%) (Fig. 1a). Other phyla with proportion greater than 1% were *Actinobacteria* (10.30%), *Bacteroidetes* (3.80%), *Chloroflexi* (3.00%), *Acidobacteria* (1.60%), *Plancomycetes* (1.10%), and *Nitrospirae* (1.10%). A total of 187 classes were identified. *Firmicutes* was mainly composed of *Clostridia* (33.60%) and *Bacilli* (16.10%). *Proteobacteria* was dominated by *Gammaproteobacteria* (10.70%), *Betaproteobacteria* (7.70%), *Alphaproteobacteria* (3.70%), and *Deltaproteobacteria* (1.70%) (Fig. 1a). However, the distribution of each taxon among the four groups was uneven, as indicated by Fig. 1b. For example, GWFG-SJL samples harboured a greater abundance of *Actinobacteria* (26.68%) than the other three groups, whereas BG-PYL samples harboured a greater abundance of *Gammaproteobacteria* (24.18%) and *Betaproteobacteria* (18.90%) among the PYL samples. The distribution of each taxon across all individuals also varied, as indicated in Fig. 1c. At the lower level, only 60.97% of sequences could be assigned to 559 genera. The dominant genera were *SMB53* (11.79%), *Lactobacillus* (10.07%), *Clostridium* (7.33%), *Faecalibacterium* (3.07%), *Solibacillus* (1.60%), *Megamonas* (1.32%), *Arthrobacter* (1.18%), and *Streptococcus* (1.15%).

### Variations in community structure of gut microbiota between two lakes and across three species

**Figure 2:**
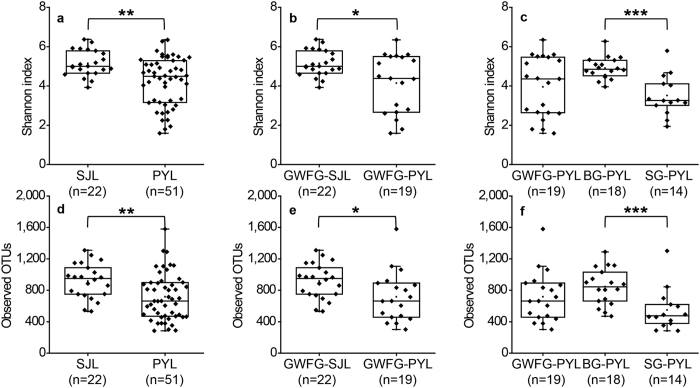
Variations of α diversity of gut microbiota in herbivorous geese. (**a**,**d**) Comparisons of Shannon index (**a**) and number of observed OTUs (**d**) between samples from Shengjin Lake (SJL) and Poyang Lake (PYL), showing *p* < 0.01 (**) difference based on Mann-Whitney test. (**b**,**e**) Comparisons of Shannon index (**b**) and number of observed OTUs (**e**) of the greater white-fronted goose (GWFG) from two lakes (SJL and PYL), showing *p* < 0.05 (*) difference based on Mann-Whitney test. (**c**,**f**) Comparisons of Shannon index (**c**) and number of observed OTUs (**f**) among samples of the three species (GWFG, Bean Goose BG and Swan Goose SG) from Poyang Lake, showing *p* < 0.001 (***) difference based on Mann-Whitney test. All panels showed interquartile range box plots (first and third quartiles) and whisker (dots indicate manes, lines medians), as well as jiggled data points.

The α diversity of the SJL samples was significantly higher than the α diversity of the PYL samples (*p* < 0.01), as indicated by the Shannon indices and number of observed OTUs (Fig. 2a,d). Among greater white-fronted goose samples, the diversity of gut microbiota was higher for GWFG-SJL than for GWFG-PYL (Fig. 2b,e, *p* < 0.05). At Poyang Lake, the gut microbiota was significantly more diverse in the BG-PYL samples than in the SG-PYL samples (*p* < 0.001), whereas α diversity of the GWFG-PYL samples did not differ significantly from those of the BG-PYL and SG-PYL samples (Fig. 2c,f).

**Figure 3:**
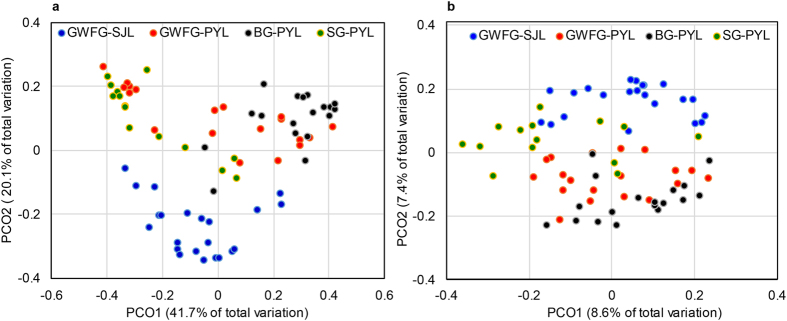
Differential gut microbial communities across all samples. (**a**) Principal coordinate analysis plot of weighted UniFrac distances for the three goose species sampled from four sites. Each point represents the gut microbiota community of an individual goose. Blue = Greater white-fronted goose from Shengjing Lake; Red = Greater white-fronted goose from Poyang Lake; Black = Bean goose from Poyang Lake; Green = Swan goose from Poyang Lake. (**b**) Principal coordinate analysis plot of unweighted UniFrac distances for the three goose species sampled from the four sites.

The differences among the four groups were also supported by patterns in β diversity. Weighted PCoA (principal coordinate analysis) revealed that samples from the same lake tended to be less different, and SJL samples clustered more closely (Fig. 3a). Statistical testing of the microbial community structure confirmed this differentiation between the two lakes (ANOSIM R = 0.169, *p* = 0.001). The bacterial community structures of GWFG-SJL and GWFG-PYL were significantly different (ANOSIM R = 0.123, *p* = 0.005). At Poyang Lake, the BG-PYL samples were significantly different from the GWFG-PYL samples (ANOSIM R = 0.342, *p* = 0.001) and SG-PYL samples (ANOSIM R = 0.118, *p* = 0.021), whereas the latter two differed less (ANOSIM R = 0.025, *p* = 0.246). Similar clustering results were obtained with unweighted PCoA (Fig. 3b).

The differences shown in α and β diversity across the four groups may derive from the contrasting abundance of each taxon. For instance, at the class level, SG-PYL samples were dominated by *Clostridium* (64.53%), while *Actinobacteria* was most abundant in GWFG-SJL samples (26.68%) (Fig. 1a). The differences were further confirmed at the genus level. The proportion of *SMB53* was lowest in BG-PYL samples (0.36%). SG-PYL samples were dominated by both *SMB53* (25.82%) and *Clostridium* (24.52%), while *Solibacillus* (3.91%) and *Arthrobacter* (2.84%) were more abundant in GWFG-SJL samples (see Supplementary Table S1).

**Figure 4:**
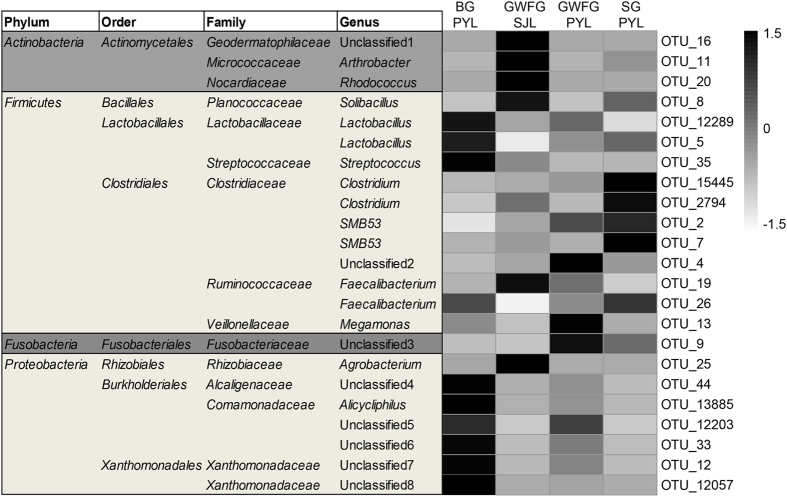
Heatmap showing the relative abundance of selected OTUs across three species from four sites. OTUs that contributed to 40% of the discrepancy between paired groups were selected using SIMPER. GWFG-SJL refers to 22 samples of greater white-fronted goose at Shengjin Lake. GWFG-PYL refers to 19 samples of greater white-fronted goose at Poyang Lake. BG-PYL refers to 18 samples of bean goose at Poyang Lake. SG-PYL refers to 14 samples of swan goose at Poyang Lake.

A heatmap of representative OTUs further confirmed this divergence (Fig. 4). All *Actinobacteria* OTUs (OTU11, OTU16 and OTU 20) were found to be more abundant in the GWFG-SJL samples. A larger proportion of *Clostridia* OTUs were detected in SG-PYL samples, which were dominated by OTU2 and OTU7. Consistent with the higher abundances of *Bacilli* and *Betaproteobacteria* in BG-PYL samples, higher abundances of OTUs of these two classes were observed in this group. For example, OTU12 and OTU33 were more abundant in BG-PYL samples. The significance tests associated with these data are presented in Supplementary Table S2.

### Distinctive pMENs across lakes and species

In addition to the numbers of species and their abundance, the complex interactions among different species are also an important component of biodiversity. In this study, we employed a random matrix theory (RMT)-based approach to construct the phylogenetic molecular ecology networks^26^ (pMENs) representing biological interactions in gut microbial communities. After basic pre-processing, 1,483, 1,427, 1,550 and 683 OTUs remained in the GWFG-SJL, GWFG-PYL, BG-PYL and SG-PYL data sets, respectively (see Supplementary Table S3). Differences were observed in terms of parameters of networks and properties of modules in GWFG between the two lakes or among the three geese species in Poyang Lake. For example, the modularity of BG-PYL group was very small (0.315, Table S3), compared to the other three groups. As a module was composed of microbes with similar ecological niches^27^, the smaller modularity of BG-PYL group suggested that microbial populations in bean goose gut might harbour higher diversity or more comprehensive interactions.

**Figure 5:**
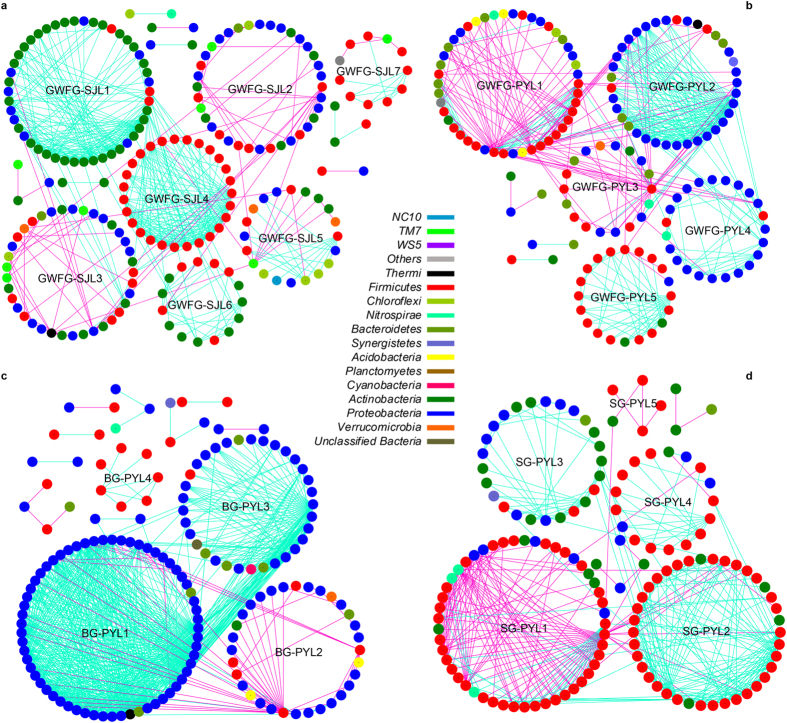
Phylogenetic Molecular Ecology Networks (pMENs) of the four groups. (**a**) The pMENs of the greater white-fronted goose from Shengjin Lake. (**b**) The pMENs of the greater white-fronted goose from Poyang Lake. (**c**) The pMENs of the bean goose from Poyang Lake. (**d**) The pMENs of the swan goose from Poyang Lake. The networks were constructed using an RMT-based model and visualized by Cytoscape 3.3.0. Nodes represent OTUs, and lines connecting nodes (edges) represent positive (light blue) and negative (light purple) interactions defined by Pearson’s correlation coefficient.

Differences were observed in terms of the overall structures and modules of pMENs constructed from SJL-GWFG and PYL-GWFG data sets. The network size of GWFG-SJL (*N* = 208 nodes, 498 links) was larger than that of GWFG-PYL (*N* = 151 nodes, 431 links). In both networks, the majority of the nodes belonged to *Proteobacteria* and *Firmicutes*, while additional *Actinobacteria* nodes (*N* = 67) were observed in the GWFG-SJL network, most of which clustered in the same module (GWFG-SJL1, Fig. 5a). In the GWFG-PYL network, nearly all *Proteobacteria* nodes clustered into two modules (GWFG-PYL2 and 4, Fig. 5b). While in GWFG-SJL, nodes of this phylum spread broadly, meaning this group of bacteria might have more diverse physiology in this network.

Networks and modules of GWFG-PYL, BG-PYL and SG-PYL were also different. Although the network size was similar among three groups, 705 links (Table S4) were constructed in the BG-PYL network with an average connectivity of 9.079, hinting more complex interactions of microbes than other two groups. In addition, 75.48% of nodes in the BG-PYL network belonged to *Proteobacteria*, nearly all of which clustered in three modules (BG-PYL1, 2 and 3, Fig. 5c). Besides, almost all the correlations between these microbes were positive, indicating the abundance of these microbes would change along the same trend. As described above, dominant composition of microbial communities of BG-PYL was *Proteobacteria*. This dominance and strong interactions of *Proteobacteria* might outcompete other microbes and lead to distinctive network of BG-PYL. This distinctiveness of community structures and microbial interactions may associate with digestibility of bean goose. However, direct evidence is still needed. SG-PYL network was dominated by *Firmicutes* and almost all of the nodes clustered in three modules, just like BG-PYL network (SG-PYL1, 2 and 4, Fig. 5d). This result indicated that *Firmicutes* microbes in swan goose gut might show greater competitiveness that others.

### Functional predictions with PICRUSt

**Figure 6:**
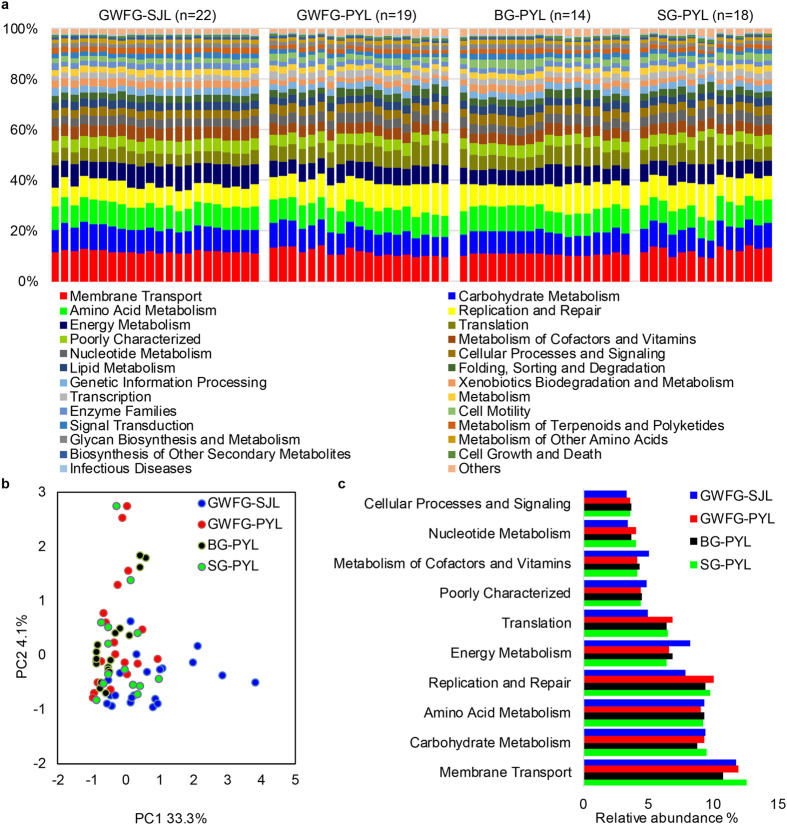
Functional predictions of all samples using PICRUSt. (**a**) Variations of functional gene families across four groups. (**b**) Principal component analysis (PCA) showing microbial functional diversity across all samples. (**c**) Histogram showing the ten dominant gene pathways across four groups. GWFG-SJL refers to 22 samples of greater white-fronted goose at Shengjin Lake. GWFG-PYL refers to 19 samples of greater white-fronted goose at Poyang Lake. BG-PYL refers to 18 samples of bean goose at Poyang Lake. SG-PYL refers to 14 samples of swan goose at Poyang Lake.

We used PICRUSt to predict changes in microbial functions that might be associated with changes in OTU abundance detected via 16S sequencing. The PICRUSt approach has been proven useful to predict genomes of organisms in environmental samples^28^ and may offer insights on the potential functions of goose gut microbiota. In this study, the chosen reference OTUs were used to match the KEGG database to predict microbial functions. Using this method, our study inferred 41 gene families in the faecal samples (Fig. 6a). We also performed PCA of the relative abundance of KEGG pathways to reveal the clustering of samples (Fig. 6b). The histogram and PCA plot both revealed that the potential functions of the microbiota of the four groups were similar (ANOSIM, *p* > 0.05).

The majority of the 41 gene families belonged to membrane transport (11.77%), carbohydrate metabolism (9.30%), amino acid metabolism (9.24%), replication and repair (8.93%), energy metabolism (7.34%), translation (5.88%), poorly characterized (4.63%), metabolism of cofactors and vitamins (4.59%), nucleotide metabolism (3.72%), and cellular processes and signalling (3.49%). The abundances of most of the gene families of GWFG-SJL differed significantly from the abundances of the other three groups in PYL, with 33 genes from GWFG-PYL, 36 from BG-PYL and 25 from SG-PYL (see Supplementary Table S5). However, the relative abundance of each gene family was more similar among the three data sets in PYL (see Supplementary Table S5). Among the ten dominant gene families noted above, the abundance of genes related to energy metabolism, carbohydrate metabolism, metabolism of cofactors and vitamins, amino acid metabolism and poorly characterized families were significantly higher in the GWFG-SJL samples than in all other data sets (Fig. 6c).

### Evaluation of the correlation between gut microbiota community structure and diet

Host species might be an important predictor of microbiota community structure because differences existed between the three species in PYL and between the three networks constructed from each data set. However, in addition to host species effects, the samples from the two lakes were also different from each other, particularly among the GWFG samples. We therefore performed diet composition analysis to further explore the mechanisms underlying these differences in microbiota community structure. Microhistological analysis revealed that for PYL individuals, almost all food was *Carex* spp., whereas in SJL, the diet was a mixture of *Poaceae* (>50%) and *Carex* spp. (<50%). We took GWFG-SJL samples to evaluate the correlation between diet composition and microbiota community structures. The Mantel test indicated that gut microbiota structures of these geese were significantly correlated with the *Carex*/*Poaceae* ratio (R = 0.304, *p* = 0.05).

## Discussion

Geese are obligate herbivores and long-distance migratory waterbirds which contribute to a variety of ecosystem services. However, little attention has been paid to the distinctive gut microbiota in geese, in contrast to the extensive studies of the abundance and distribution of these birds. In this research, the community composition and structure of the gut microbiota of wintering greater white-fronted, bean and swan geese were explored. We observed significant variations of microbial composition between the same species at two lakes and among the three species at the same lake. Differences in microbial community structures and interactions were also identified. Functional analysis showed that diverse gut microbiota performed similar functions despite differences in taxonomic composition.

The gut microbiota in the three goose species were dominated by *Firmicutes, Proteobacteria* and *Actinobacteria*. However, the detailed composition of these phyla at lower taxonomic levels was notably altered in different groups. In the SG-PYL group, *Firmicutes* was mostly composed of *Clostridia* at the class level and *Clostridium* (especially *Clostridium celatum*) at the genus level. *Clostridium celatum* was first discovered in normal human faeces, and it is associated with production of acetic and formic acids^29^. *Clostridium celatum* also assists in the metabolism of soy isoflavones, which is important for consumption of high-protein soy^30^. Among the three geese species, swan goose shows the largest body size which entails a higher energy requirement^19^. Besides, swan geese are supposed to feed on high-protein food, such as tubers of *Vallisneria natans*, to get sufficient energy^23^. Thus, the enrichment of *Firmicutes* in the SG-PYL samples may contribute to energy intake and nutrient absorption of swan geese. However, the detailed roles that these microbes perform in swan goose await further clarification.

Significant differences in the distribution of *Proteobacteria* were also discovered, especially the enrichment of *Gammaproteobacteria* (24.18%) and *Betaproteobacteria* (18.90%) in bean goose samples. The great abundances of these two classes were due largely to families of *Xanthomonadaceae* and *Comamonadaceae* which used to be found in gut microbiota of black-legged kittiwakes^31^, *Procellariiformes* seabirds^11^ and Canada goose^17^. These two families are strong competitors with flexible metabolism and are considered essential for host’s digestion of nutritionally poor diet^32^. Some species of these families may exhibit cellulase activity and degrade a variety of aromatic compounds^33^. Therefore, we hypothesized that the enrichment of these families may facilitate cellulose degradation and nutrient absorption for bean goose. Although cellulose digestion was found in other geese^34^, there is no direct evidence of cellulose digestion in bean goose which means our hypothesis awaits further confirmation. Another significant difference was the high abundance of *Actinobacteria* in greater white-fronted goose at Shengjin Lake, primarily due to the predominance of *Geodermatophilaceae* (2.04%), *Micrococcaceae* (2.84%) and *Nocardiaceae* (1.87%). As most members of these families are present in soil, water or the air^35^, these bacteria may have originated from the environment, implying horizontal transmission could also influence the structure of animal gut microbiota^36^. Whether these bacteria perform certain functions for the host remains unclear.

Considering factors shaping and shifting gut microbial structure, genetics (host species) is one of the most important ones^37^. The coexistence of three geese species at the same lake (Poyang Lake) allowed us to test the effect of host species on goose gut microbiota. In addition to differences in the distributions of dominant phyla shown above, significant divergences of microbial community structures were identified, as reflected in terms of α diversity, UniFrac clustering and microbial interactions. The microbial community structure of bean goose samples was significantly different from those of the greater white-fronted and swan geese, whereas the latter two groups were less different. These differences might reflect the ability of bean goose to feed on more diverse food resources than the other two species^22^, although only *Carex* spp. was present in the microhistological analysis of droppings at Poyang Lake in this study. The results suggest that host species can be a strong predictor of goose gut microbiota, in accordance with previous studies demonstrating the significance of vertical transmission in shaping host gut microbial structure^38^.

Diet has also been recognized as an important driver of gut microbial community structure, such as the distinctive clustering of carnivores, herbivores and omnivores^39^. Our results also demonstrated substantial effects of diet on changes in the geese gut microbial structures, as reflected by the significant differences between the greater white-fronted geese from two lakes. In fact, the discrepancy in diets between the two lakes was small; at Poyang Lake, GWFG fed almost exclusively on *Carex* spp., whereas at Shengjin Lake, an extra component of *Poaceae* was identified. Both of these species are typical monocots present in wetlands and fed upon by diverse geese. Whereas tissues of *Carex* spp. contain relatively more fibre, leaves of *Poaceae* tend to be softer and more palatable^25^. The obvious differentiation of gut microbial structures may indicate high sensitivity of gut microbiota to changes in food^40^. The gut microbiota of herbivores is traditionally thought to participate in the digestion of cellulose or other dietary fibre, resulting in profound interactions between microbes and diets^41^. However, only small amounts of cellulose digestion- or fermentation-associated bacteria were observed in our study. The shortage of such microbes might be related to the high energy requirements and high metabolism rate of geese. The process of food digestion by herbivorous geese is dominated by gizzard mastication and acid hydrolysis, enabling rapid uptake of soluble carbohydrate and protein derived from the selective cropping of high quality foods, whereas microbe-dependent fermentation is traditionally thought to contribute relatively little in supplying energy^42^.

The diverse microbes in geese gut may present many important functions which are essential to geese life. In this study, we employed the PICRUSt to infer potential gene profiles from 16S rRNA sequencing. The results indicated that the most abundant functional categories were associated with membrane transport, carbohydrate metabolism, amino acid metabolism, replication and repair, and energy metabolism. The importance of carbohydrate metabolism and amino acid metabolism used to be predicted in a meta-analysis of avian microbiota using PICRUSt^6^. However, the interpretation of the predictive results should be cautious due to the inherent limitations of PICRUSt. For example, the prediction is dependent on reference genomes which are phylogenetically similar to those presented in a community^28^. As the reference genome sequencing of geese gut microbiota was not as intensive as other communities (such as the human microbiota), the prediction accuracy of PICRUSt in geese needs further validation. Besides, PICRUSt could only handle OTUs which were matched to available database, while missed the novel, unstudied OTUs^28^. Even though some functions could be inferred using predictive methods such as PICRUSt, many of the actual functions of the gut microbiota still deserve to be uncovered, with the help of multiple omics approaches^43^.

The microbial community structures and functions are influenced not only by their taxonomy, abundance, but also by microbial interactions^27^. In this study, we tried to reveal interactions of geese gut microbiota by constructing pMENs. We found clear modules in four groups, indicating that gut microbiota in geese were not assembled randomly. Obvious differences were observed in terms of overall structures and properties of networks. This phenomenon was consistent with variations of community composition and structures. Thus, it is reasonable to infer that microbial interactions are associated with community structures. With the potential to outcompete other microbes and show positive correlations, the dominant phyla shown in network may have profound influences on the microbial functions^27^. However, as this kind of interactions are based on 16S rRNA sequencing, the actual interactions may await for more efforts.

## Conclusion

We depicted the basic composition of gut microbiota in the greater white-fronted, bean and swan geese at Shengjin Lake and Poyang Lake during wintering period. These geese shared dominant phyla of *Firmicutes, Proteobacteria* and *Actinobacteria*. Functional predictions indicated that these microbes may assist in the energy intake and nutrient absorption for these birds. Our study also demonstrated potential influences of the host species and diet on the microbial community assemblages with significant variations among the four groups. Yet, non-invasive sampling of faeces entails a risk that our samples may be unrepresentative of the age, sex or health status, factors that potentially drive animal gut microbiota composition. Thus, more efforts are needed to fully understand the gut microbiota in geese population as a whole. Besides, whether and how these microbial organisms interact directly with geese remains a question. Despite these, our study provided a helpful reference for future researches which may incorporate multiple methods to deeply investigate the gut microbiota of herbivorous geese and other waterbirds.

## Materials and Methods

### Ethical approval

There was no need for an ethical statement because only faeces of geese were collected for DNA extraction and relevant molecular studies. No direct capture or hunting involved. Lake access was permitted by the Anhui Shengjin Lake National Nature Reserve Administration and Jiangxi Nanji Wetland National Nature Reserve Administration, agencies which are responsible for the protection and management of these two lakes.

### Sampling

For this study, 73 goose faeces were sampled from Shengjin Lake (SJL, *N* = 22) and Poyang Lake (PYL, *N* = 51). At SJL, all samples belonged to GWFG (GWFG-SJL). In PYL, we collected 19 GWFG samples (GWFG-PYL), 18 BG samples (BG-PYL) and 14 SG samples (SG-PYL). We chose sampling sites where only a single species was present based on synchronous waterbird surveys and waited while geese were feeding^23^. Fresh droppings were collected and stored in sterile tubes. To avoid cross-contamination, disposable gloves were changed for each sample. To ensure that all samples belonged to different individuals, the samples were collected at a minimum distance interval of two meters. All samples were transported to the laboratory on dry ice and stored at −80 °C until further treatment.

### DNA extraction, PCR amplification, and high-throughput sequencing

DNA was extracted from the faeces using a modified CTAB protocol^44^ with a minor modification in incubation time (extended to 12 h). To recover rare species, two replicates were performed and subsequently pooled for each sample. For each batch of DNA extractions, negative controls (i.e., extraction without faeces) were included to monitor possible contamination. The DNA extracts were used as templates to amplify the V4 region of 16S rRNA gene with primers of 515F and 806R^45^. The primers for each sample were modified with an addition of twelve nucleotide tag at the 5′ end to distinguish all samples. Amplification was performed in a total volume of 25 μl, and three replicates were performed for each sample. Each replicate consisted of 100 ng of DNA template, 1 U of Taq polymerase (Takara, Dalian, Liaoning Prov., China), 1 × buffer, 2 mM MgCl_2_, 0.25 mM each dNTP, and 0.1 μM forward and reverse primers, respectively. After 4 min of denaturation at 94 °C, 35 cycles of 30 s at 94 °C, 30 s at 56 °C, and 45 s at 72 °C were performed, followed by a final elongation for 10 min at 72 °C. Three replicates of each sample were pooled and purified using Sangon PCR product gel extraction kit (Sangon Biotech, Shanghai, China). Quantification was performed with a NanoDrop ND-2000 UV-Vis spectrophotometer (NanoDrop Technologies, Delaware, United States of America). High-throughput sequencing was performed using the Illumina MiSeq platform following manufacturer’s instructions at Central South University, Changsha, China.

### Diet analysis via microhistological observation

We used the method of Zhang to perform microhistological observation^23^. Each sample was first washed with pure water and filtered with a 25-μm filter. The suspension was subsequently examined under a light microscope at 10 × magnification for quantification statistics and at 40 × magnification for species identification. We compared photos of visible fragments with previous epidermis database to identify food items.

### Sequence analysis and statistical analysis

Amplicons were sequenced using the Illumina Miseq platform at the Central South University, Changsha, China. Galaxy (http://mem.rcees.ac.cn:8080/) was used for sequencing data analysis unless otherwise noted. Raw data was sorted into independent files according to unique tags. After removal of tags and primers, pair-ended sequences were merged. Quality filtering included three steps: we discarded sequences (i) containing “N”; (ii) with quality score less than 20 (<Q20); (iii) with length shorter than 200 bp. All sequences were classified into OTUs at a threshold of 97% similarity using UPARSE pipeline^46^ and representative sequences of each OTU were generated simultaneously. Taxonomic assignment of representative sequences were performed with the Ribosomal Database Project (RDP) naïve Bayesian rRNA classifier at an 80% confidence level against a Greengenes reference taxonomy (Greengenes 13.8)^47^. Sequences identified as *Achaea, Chloroplast* and *Mitochondria* were excluded from subsequent analysis. The following alpha diversity metrics were calculated: Shannon diversity metric and observed OTUs metric. Rarefaction curves were generated with R “vegan” package^48^. Representative sequences were then aligned using PyNAST with reference sequences from 16S Greengenes^49^ and a phylogenetic tree was constructed with FastTree^50^,^51^.

Resampled OTU table (10,000 sequences per sample) was used to calculate the α diversity (Shannon index and number of observed OTUs). UniFrac was employed to compare β diversity with the phylogenetic tree and the resampled OTU table^52^. The Mann-Whitney test was used for univariate statistical analysis. All univariate statistical analysis was conducted using PRIMER 5 (PRIMER-E Ltd., Lutton, UK) and SPSS 21.0 (International Business Machines Corp., Armonk, NY). ANOSIM was performed to identify significant differences in gut microbiota structure using the method implemented in the R “vegan” package^48^. The Mantel test was used to detect the correlation between diet composition and microbial community structures with the R “vegan” package^48^. In this study, we used SIMPER in PRIMER 5 to filter out OTUs that contributed to 40% of dissimilarity between each two groups and these OTUs were selected as representatives which were used to produce a heatmap with “pheatmap” in R.

### Molecular ecology network analyses

Bacteria in the same environment tends to co-occur, sharing similar niches or performing certain functions, rather than assembles randomly. The random matrix theory (RMT)-based approach is a way to construct phylogenetic molecular ecology networks (pMENs) which will represent biological interactions in microbial communities^26^. Positive interactions/correlations hypothetically indicate symbiosis or mutualism, while negative interactions/correlations may signal competition or parasitism. In this study, OTUs with >10 sequences in each group were retained for network construction, using the authors’ website (http://129.15.40.240/mena/main.cgi). The networks were constructed using RMT models, after taking logarithm and Pearson correlation estimation^27^. Complementary network properties were also calculated to describe network differences, such as connectivity (which means the number of links of one node to other nodes), modularity (which measures the degree to which the network was organized into clearly delimited modules), geodesic distance (which is the shortest path between two nodes), and clustering coefficient (which indicates how well a node is connected with its neighbours). The networks were visualized using Cytoscape 3.3.0^53^.

### Functional predictions using PICRUSt

PICRUSt is a bioinformatics tool that uses marker genes to predict the functional content of microorganism^28^. In this study, this method was employed to predict the potential functions of each sample based on 16S rRNA sequencing data. We used the KEGG database and performed closed reference OTU picking using the sampled reads against a Greengenes reference taxonomy (Greengenes 13.5). The 16S copy number was then normalized, molecular functions were predicted and final data were summarized into KEGG pathways. The differences in predicted molecular functions of bacterial communities among four groups were shown by PCA (principal component analysis) using R “vegan” package. ANOSIM was used to test whether the dissimilarity of gene abundance among the four groups was significant. All PICRUSt analyses were conducted online (http://mem.rcees.ac.cn:8080/root).

## Additional Information

**Accession Code**: Raw data of sequencing have been uploaded to GenBank SRA. The accession number is SRP078554.

**How to cite this article**: Yang, Y. *et al*. Characterising the interspecific variations and convergence of gut microbiota in *Anseriformes* herbivores at wintering areas. *Sci. Rep.* **6**, 32655; doi: 10.1038/srep32655 (2016).

## Supplementary Material

Supplementary Information
