# Supplementary material for: Characterising the interspecific variations and convergence of gut microbiota in Anseriformes herbivores at wintering areas
Source: Sci Rep. 2016 Sep 7;6:32655. doi: 10.1038/srep32655 (PMC5013396; doi:10.1038/srep32655)
Supplement: Supplementary Information [file srep32655-s1.pdf]

Supplementary information for:

Characterising the interspecific variations and convergence of gut microbiota in *Anseriformes* herbivores at wintering areas

Yuzhan Yang[1], Ye Deng[2], Lei Cao[2,*]

[1]School of Life Sciences, University of Science and Technology of China, Huangshan Road, Hefei, China, 230026

[2]Research Center of Eco-Environmental Sciences, Chinese Academy of Sciences, Beijing, China, 100085

Correspondence and requests for materials should be addressed to L.C. (email: caolei@ustc.edu.cn)

Fig. S1 Rarefaction curves of all samples. The eight panels correspond to OTU-level and Shannon index rarefaction curves of the greater white-fronted goose samples (n=22) at Shengjin Lake (a, e); the greater white-fronted goose samples (n=19) at Poyang Lake (b, f); the bean goose samples (n=18) at Poyang Lake (c, g); and the swan goose samples (n=14) at Poyang Lake (d, h), respectively.

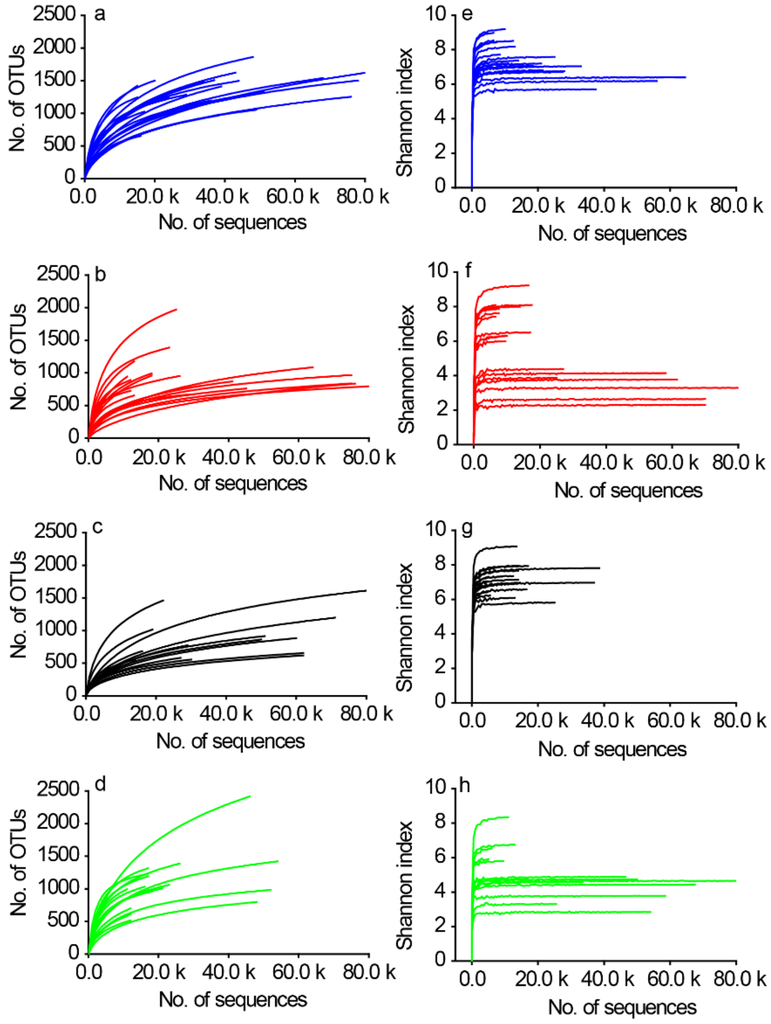

Table S1. Composition of dominant genera across the four groups. GWFG-SJL refers to 22 samples of greater white-fronted goose at Shengjin Lake. GWFG-PYL refers to 19 samples of greater white-fronted goose at Poyang Lake. BG-PYL refers to 18 samples of bean goose at Poyang Lake. SG-PYL refers to 14 samples of swan goose at Poyang Lake.

Genus	GWFG-SJL	GWFG-PYL	BG-PYL	SG-PYL
Unclassified	36.30	44.67	53.15	18.31
SMB53	7.81	16.27	0.36	25.82
Lactobacillus	8.03	10.95	12.41	9.10
Clostridium	4.42	4.14	0.37	24.52
Faecalibacterium	2.02	3.55	3.21	3.84
Solibacillus	3.91	0.04	0.00	2.11
Megamonas	0.12	3.32	1.21	0.58
Arthrobacter	2.84	0.37	0.33	0.81
Streptococcus	1.52	0.33	2.21	0.39
Rothia	1.47	0.78	0.92	0.56
Agrobacterium	2.65	0.05	0.31	0.06
Epulopiscium	1.04	0.30	0.01	2.35
Acidithiobacillus	1.01	0.50	1.24	0.43
Leptospirillum	0.60	0.74	0.76	0.95
Alicycliphilus	0.08	0.48	2.12	0.01
Bacillus	1.37	0.14	0.09	0.85
Sulfobacillus	0.40	0.62	0.88	0.66
Shewanella	0.23	0.73	0.78	0.69
Rhodococcus	1.91	0.01	0.03	0.01
Pseudoalteromonas	0.24	0.57	0.78	0.59
Prevotella	1.14	0.29	0.31	0.09
Turicibacter	1.00	0.06	0.14	0.58
Nocardioides	1.50	0.01	0.01	0.03
Pseudomonas	0.46	0.44	0.64	0.18
Paenibacillus	1.04	0.01	0.00	0.40
Lactococcus	0.44	0.23	0.69	0.02
vadinCA02	0.31	0.13	0.91	0.01
Flavobacterium	0.30	0.34	0.45	0.24
Helicobacter	0.44	0.21	0.37	0.20
Bacteroides	0.13	0.40	0.64	0.08

Mycobacterium	0.69	0.11	0.12	0.10
Geobacter	0.09	0.14	0.88	0.04
Exiguobacterium	0.67	0.01	0.01	0.36
Acinetobacter	0.21	0.30	0.42	0.17
Oscillospira	0.15	0.39	0.35	0.19
Butyricicoccus	0.03	0.30	0.71	0.04
Terracoccus	0.78	0.02	0.02	0.07
Vibrio	0.11	0.34	0.31	0.29
Bifidobacterium	0.84	0.00	0.00	0.00
Janthinobacterium	0.33	0.23	0.25	0.16
Others	11.35	7.43	11.59	4.14
Total	100.00	100.00	100.00	100.00

Table S2. Significance test (*p* value) of selected OTUs between paired groups. OTUs that contributed to 40% of the discrepancy between paired groups were selected. GWFG-SJL refers to 22 samples of greater white-fronted goose at Shengjin Lake. GWFG-PYL refers to 19 samples of greater white-fronted goose at Poyang Lake. BG-PYL refers to 18 samples of bean goose at Poyang Lake. SG-PYL refers to 14 samples of swan goose at Poyang Lake. The Mann-Whitney test was used for univariate statistical analysis.

ID	GWFG-SJL vs GWFG-PYL	GWFG-SJL vs BG-PYL	GWFG-SJL vs SG-PYL	GWFG-PYL vs BG-PYL	GWFG-PYL vs SG-PYL	BG-PYL vs SG-PYL
OTU_2794	0.000	0.000	0.077	0.000	0.000	0.000
OTU_8	0.000	0.000	0.038	0.022	0.000	0.000
OTU_33	0.000	0.000	0.001	0.004	0.000	0.000
OTU_13885	0.012	0.000	0.000	0.004	0.000	0.000
OTU_4	0.834	0.000	0.399	0.001	0.870	0.000
OTU_12203	0.000	0.000	0.336	0.121	0.000	0.000
OTU_12057	0.000	0.000	0.155	0.001	0.000	0.000
OTU_12	0.000	0.000	0.000	0.002	0.000	0.000
OTU_44	0.037	0.000	0.000	0.001	0.000	0.000
OTU_2	0.958	0.000	0.009	0.000	0.244	0.000
OTU_15445	0.824	0.000	0.005	0.000	0.012	0.000
OTU_7	0.003	0.000	0.091	0.031	0.004	0.000
OTU_35	0.019	0.362	0.015	0.001	0.770	0.003
OTU_11	0.000	0.000	0.000	0.988	0.018	0.014
OTU_12289	0.239	0.765	0.115	0.504	0.026	0.068
OTU_9	0.000	0.038	0.000	0.003	0.125	0.078
OTU_25	0.000	0.000	0.000	0.000	0.024	0.081
OTU_20	0.000	0.000	0.000	0.563	0.414	0.165
OTU_16	0.000	0.000	0.000	0.159	0.939	0.175
OTU_26	0.016	0.263	0.724	0.683	0.106	0.583
OTU_13	0.000	0.013	0.109	0.167	0.083	0.598
OTU_19	0.087	1.000	0.578	0.168	0.053	0.695
OTU_5	0.302	0.744	0.721	0.504	0.870	0.849

Table S3. Parameters' comparisons of pMENs (phylogenetic molecular ecology networks) of four groups. GWFG-SJL refers to 22 samples of greater white-fronted goose at Shengjin Lake. GWFG-PYL refers to 19 samples of greater white-fronted goose at Poyang Lake. BG-PYL refers to 18 samples of bean goose at Poyang Lake. SG-PYL refers to 14 samples of swan goose at Poyang Lake.

Group	GWFG-SJL	GWFG-PYL	BG-PYL	SG-PYL
No. of original OTUs	1483	1427	1550	683
Modularity (no. of modules)	0.681(14)	0.560(8)	0.315(13)	0.572(7)
Modules with >5 nodes	7	5	4	5
Network size (total nodes)	208	151	155	141
Total links	498	431	705	371
Average Connectivity	4.788	5.709	9.097	5.262
Average clustering coefficient	0.258	0.276	0.302	0.250
Average geodesic distance	3.986	3.481	2.068	4.067
Nodes with max degree	OTU20	OTU15085	OTU12057	OTU6973
Nodes with max betweenness	OTU588	OTU8242	OTU6973	OTU6973
Nodes with max stress centrality	OTU103	OTU7908	OTU6973	OTU6973
Nodes with max eigenvector centrality	OTU8	OTU12	OTU12057	OTU2794
Similarity threshold	0.730	0.690	0.830	0.790

Table S4. Comparisons of module composition, module size and microbial interactions of pMENs (phylogenetic molecular ecology networks). GWFG-SJL refers to 22 samples of greater white-fronted goose at Shengjin Lake. GWFG-PYL refers to 19 samples of greater white-fronted goose at Poyang Lake. BG-PYL refers to 18 samples of bean goose at Poyang Lake. SG-PYL refers to 14 samples of swan goose at Poyang Lake.

Module composition				
Phylum	GWFG-SJL	GWFG-PYL	BG-PYL	SG-PYL
NC10	1	0	0	0
Nitrospirae	1	3	1	3
Tenericutes	1	1	0	0
Thermi	1	1	1	0
Bacteroidetes	2	16	8	2
TM7	2	0	0	0
Verrucomicrobia	3	1	1	0
Acidobacteria	6	3	2	0
Chloroflexi	7	2	0	0
Proteobacteria	50	67	117	19
Actinobacteria	67	6	1	25
Firmicutes	67	50	21	91
Synergistetes	0	1	1	0
Cyanobacteria	0	0	1	0
Unclassified	0	0	1	1
Total	208	151	155	141
Module size (Number of nodes)				
Module (with ⩾ 5 nodes)	GWFG-SJL	GWFG-PYL	BG-PYL	SG-PYL
1	46	46	61	49
2	36	37	32	38
3	37	18	33	25
4	27	23	7	17
5	20	20	-	5
6	15	-	-	-
7	10	-	-	-
Microbial interactions				
Interactions	GWFG-SJL	GWFG-PYL	BG-PYL	SG-PYL

Positive	392	280	649	249
Negative	106	151	56	122

Table S5. Significance test of KEGG pathways between paired groups. GWFG-SJL refers to 22 samples of greater white-fronted goose at Shengjin Lake. GWFG-PYL refers to 19 samples of greater white-fronted goose at Poyang Lake. BG-PYL refers to 18 samples of bean goose at Poyang Lake. SG-PYL refers to 14 samples of swan goose at Poyang Lake. The Mann-Whitney test was used for univariate statistical analysis.

KEGG pathways	GWFG-SJL vs GWFG-PYL	GWFG-SJL vs BG-PYL	GWFG-SJL vs SG-PYL	GWFG-PYL vs BG-PYL	GWFG-PYL vs SG-PYL	BG-PYL vs SG-PYL
Amino Acid Metabolism	0.003	0.000	0.041	0.504	0.325	0.171
Biosynthesis of Other Secondary Metabolites	0.000	0.000	0.003	0.429	0.344	0.138
Cancers	0.513	0.115	0.516	0.362	0.942	0.287
Carbohydrate Metabolism	0.004	0.000	0.038	0.378	0.444	0.095
Cardiovascular Diseases	0.050	0.036	0.256	0.952	0.344	0.254
Cell Communication	0.409	0.130	0.000	0.622	0.041	0.054
Cell Growth and Death	0.050	0.003	0.119	0.346	0.560	0.111
Cell Motility	0.039	0.039	0.314	0.605	0.536	0.676
Cellular Processes and Signaling	0.012	0.003	0.085	0.564	0.444	0.239
Circulatory System	0.017	0.003	0.163	0.605	0.344	0.171
Digestive System	0.000	0.000	0.000	0.121	0.971	0.074
Endocrine System	0.000	0.000	0.000	0.605	0.444	0.704
Energy Metabolism	0.000	0.000	0.004	0.362	0.344	0.087
Environmental Adaptation	0.403	0.054	0.770	0.412	0.423	0.068
Enzyme Families	0.001	0.000	0.014	0.466	0.362	0.074
Excretory System	0.000	0.000	0.000	0.485	0.145	0.518

Folding, Sorting and Degradation	0.019	0.001	0.092	0.362	0.636	0.081
Genetic Information Processing	0.002	0.000	0.030	0.447	0.382	0.087
Glycan Biosynthesis and Metabolism	0.003	0.003	0.009	0.715	0.489	0.494
Immune System	0.448	0.021	0.922	0.191	0.382	0.048
Immune System Diseases	0.000	0.000	0.003	0.808	0.716	0.543
Infectious Diseases	0.002	0.000	0.012	0.378	0.560	0.119
Lipid Metabolism	0.002	0.001	0.012	0.671	0.402	0.425
Membrane Transport	0.006	0.000	0.085	0.346	0.308	0.063
Metabolic Diseases	0.000	0.000	0.003	0.191	0.291	0.025
Metabolism	0.001	0.000	0.012	0.412	0.344	0.210
Metabolism of Cofactors and Vitamins	0.001	0.000	0.006	0.485	0.325	0.068
Metabolism of Other Amino Acids	0.000	0.000	0.003	0.976	0.362	0.621
Metabolism of Terpenoids and Polyketides	0.000	0.000	0.002	0.693	0.382	0.403
Nervous System	0.001	0.000	0.056	0.162	0.135	0.007
Neurodegenerative Diseases	0.347	0.232	0.795	0.952	0.536	0.470
Nucleotide Metabolism	0.036	0.001	0.243	0.288	0.402	0.068
Poorly Characterized	0.002	0.000	0.021	0.564	0.382	0.305
Replication and Repair	0.089	0.003	0.475	0.302	0.362	0.058
Sensory System	0.645	0.337	0.000	0.644	0.022	0.031
Signal Transduction	0.001	0.001	0.006	0.715	0.382	0.649
Signaling Molecules and Interaction	0.001	0.001	0.011	0.761	0.771	0.676
Transcription	0.039	0.001	0.721	0.261	0.259	0.017

Translation	0.229	0.011	0.721	0.288	0.536	0.058
Transport and Catabolism	0.001	0.001	0.003	0.855	0.689	0.879
Xenobiotics Biodegradation and Metabolism	0.000	0.000	0.001	0.412	0.536	1.000
